# Supplementary material for: Gi/o protein-coupled receptors in dopamine neurons inhibit the sodium leak channel NALCN
Source: eLife. 2018 Dec 17;7:e40984. doi: 10.7554/eLife.40984 (PMC6305199; doi:10.7554/eLife.40984)
Supplement: Transparent reporting form [file elife-40984-transrepform.docx]

***eLife’s* transparent reporting form**

We encourage authors to provide detailed information *within their submission* to facilitate the interpretation and replication of experiments. Authors can upload supporting documentation to indicate the use of appropriate reporting guidelines for health-related research (see [EQUATOR Network](http://www.equator-network.org/%20)), life science research (see the [BioSharing Information Resource](https://biosharing.org/)), or the [ARRIVE guidelines](http://www.plosbiology.org/article/info:doi/10.1371/journal.pbio.1000412) for reporting work involving animal research. Where applicable, authors should refer to any relevant reporting standards documents in this form.

If you have any questions, please consult our Journal Policies and/or contact us: [editorial@elifesciences.org](mailto:editorial@elifesciences.org).

**Sample-size estimation**

- You should state whether an appropriate sample size was computed when the study was being designed
- You should state the statistical method of sample size computation and any required assumptions
- If no explicit power analysis was used, you should describe how you decided what sample (replicate) size (number) to use

Please outline where this information can be found within the submission (e.g., sections or figure legends), or explain why this information doesn’t apply to your submission:

Although we report the exact p-values to four decimal places for all of our results, sample size was not computed when the study was designed. However, we performed post hoc power analysis (using GPower 3.1) for comparisons that reached statistical significance (p<0.05). Most of our positive findings fall above a 0.9 power value. However, in one case in which we found statistically significant values (p<0.05), we found that statistical power fell below 0.8. The significant difference (p=0.0078) between the isolated sodium leak current in 2 mM calcium before and after sodium substitution by NMDG (Fig.1C) shows a power of 0.69.

**Replicates**

- You should report how often each experiment was performed
- You should include a definition of biological versus technical replication
- The data obtained should be provided and sufficient information should be provided to indicate the number of independent biological and/or technical replicates
- If you encountered any outliers, you should describe how these were handled
- Criteria for exclusion/inclusion of data should be clearly stated
- High-throughput sequence data should be uploaded before submission, with a private link for reviewers provided (these are available from both GEO and ArrayExpress)

Please outline where this information can be found within the submission (e.g., sections or figure legends), or explain why this information doesn’t apply to your submission:

Only experiments in which all solutions could be applied are included and outliers were not omitted under any conditions. In the different figures, all data from individual cells are shown (except for the normalized firing rate in Fig.3 and 5, to allow a better readability). The holding current values were obtained by averaging the last 10 seconds in each condition. At least 3 animals were tested per condition.

**Statistical reporting**

- Statistical analysis methods should be described and justified
- Raw data should be presented in figures whenever informative to do so (typically when N per group is less than 10)
- For each experiment, you should identify the statistical tests used, exact values of N, definitions of center, methods of multiple test correction, and dispersion and precision measures (e.g., mean, median, SD, SEM, confidence intervals; and, for the major substantive results, a measure of effect size (e.g., Pearson's r, Cohen's d)
- Report exact p-values wherever possible alongside the summary statistics and 95% confidence intervals. These should be reported for all key questions and not only when the p-value is less than 0.05.

Please outline where this information can be found within the submission (e.g., sections or figure legends), or explain why this information doesn’t apply to your submission:

Statistical significance was determined in 2 group comparisons by two-tailed Mann-Whitney U-test or Wilcoxon signed-rank test (paired comparisons) and in more than 2 groups comparisons by one-way ANOVAs or one-way repeated measures ANOVAs (paired comparisons) followed by the Tukey’s post hoc test. Raw data values from individual cells are always plotted when averaged data are provided (except for the normalized firing rate in Fig.3 and 5). This information is clearly stated in the Results section as well as in the materials and methods. A table describing all statistical tests described in the paper is appended to this worksheet.

(For large datasets, or papers with a very large number of statistical tests, you may upload a single table file with tests, Ns, etc., with reference to sections in the manuscript.)

**Group allocation**

- Indicate how samples were allocated into experimental groups (in the case of clinical studies, please specify allocation to treatment method); if randomization was used, please also state if restricted randomization was applied
- Indicate if masking was used during group allocation, data collection and/or data analysis

Please outline where this information can be found within the submission (e.g., sections or figure legends), or explain why this information doesn’t apply to your submission:

Samples were allocated into the different experimental groups based on the genotype of the mice (either TH-GFP or NALCN knock-out).

**Additional data files (“source data”)**

- We encourage you to upload relevant additional data files, such as numerical data that are represented as a graph in a figure, or as a summary table
- Where provided, these should be in the most useful format, and they can be uploaded as “Source data” files linked to a main figure or table
- Include model definition files including the full list of parameters used
- Include code used for data analysis (e.g., R, MatLab)
- Avoid stating that data files are “available upon request”

Please indicate the figures or tables for which source data files have been provided:

| **Comparison** | **Figure** | **n** | **statistical test** | **p-Value** | **power** |  |
| --- | --- | --- | --- | --- | --- | --- |
| WT V-clamp 2mM Calcium vs NMDG | 1C | 8 | Wilcoxon test | p=0.0078 | 0.69 |  |
| WT V-Clamp NMDG 0.1 mM Calcium | 1F | 22 | RM-ANOVA | p<0.0001 | 1 |  |
| WT V-Clamp 2 vs 0.1 mM calcium | 1F | 22 | Tukey post hoc | p<0.0001 |  |  |
| WT V-Clamp 0.1 mM calcium vs NMDG | 1F | 22 | Tukey post hoc | p<0.0001 |  |  |
| KO V-Clamp NMDG 0.1 mM Calcium | 1I | 9 | RM-ANOVA | p=0.0164 | 1 |  |
| KO V-Clamp 2 vs 0.1 mM calcium | 1I | 9 | Tukey post hoc | p=0.0236 |  |  |
| KO V-Clamp 0.1 mM calcium vs NMDG | 1I | 9 | Tukey post hoc | p=0.0531 |  |  |
| WT V-clamp dopamine 0.1 mM Calcium | 2A | 14 | RM-ANOVA | p<0.0001 | 1 |  |
| WT V-Clamp 2 vs 0.1 mM calcium | 2A | 14 | Tukey post hoc | p<0.0001 |  |  |
| WT V-Clamp 0.1 mM calcium vs dopamine | 2A | 14 | Tukey post hoc | p<0.0001 |  |  |
| WT V-Clamp 0.1 mM dopamine vs NMDG | 2A | 14 | Tukey post hoc | p=0.4319 |  |  |
| KO V-clamp dopamine 0.1 mM Calcium | 2B | 7 | RM-ANOVA | p=0.0025 | 1 |  |
| KO V-Clamp 2 vs 0.1 mM calcium | 2B | 7 | Tukey post hoc | p=0.0717 |  |  |
| KO V-Clamp 0.1 mM calcium vs dopamine | 2B | 7 | Tukey post hoc | p=0.1857 |  |  |
| KO V-Clamp 0.1 mM dopamine vs NMDG | 2B | 7 | Tukey post hoc | p=0.9987 |  |  |
| B-arrestin KO V-clamp dopamine 0.1 Calcium | 2D | 6 | RM-ANOVA | p<0.0001 | 1 |  |
| B-arrestin KO V-Clamp 2 vs 0.1 mM calcium | 2D | 6 | Tukey post hoc | p=0.0008 |  |  |
| B-arrestin KO V-Clamp 0.1 mM calcium vs dopamine | 2D | 6 | Tukey post hoc | p=0.012 |  |  |
| B-arrestin KO V-Clamp 0.1 mM dopamine vs NMDG | 2D | 6 | Tukey post hoc | p=0.0163 |  |  |
| WT V-clamp dopamine GDP-BS 2 mM calcium | 2E | 8 | RM-ANOVA | p=0.0002 | 1 |  |
| WT V-clamp dopamine GDP-BS 2 mM calcium 15 min | 2E | 8 | Tukey post hoc | p=0.0027 |  |  |
| WT V-clamp dopamine GDP-BS 2 mM calcium 15 min vs DA | 2E | 8 | Tukey post hoc | p=0.4196 |  |  |
| WT V-clamp dopamine GDP-BS DA vs NMDG | 2E | 8 | Tukey post hoc | p=0.0037 |  |  |
| WT V-clamp dopamine GDP-BS 0.1 mM calcium | 2F | 5 | RM-ANOVA | p=0.0001 | 0.99 |  |
| WT V-clamp dopamine GDP-BS 2 vs 0.1 mM calcium | 2F | 5 | Tukey post hoc | p=0.0006 |  |  |
| WT V-clamp dopamine GDP-BS 0.1 mM calcium vs DA | 2F | 5 | Tukey post hoc | p=0.9992 |  |  |
| WT V-clamp dopamine GDP-BS DA vs NMDG | 2F | 5 | Tukey post hoc | p=0.0010 |  |  |
| Dopamine sensitive current | 2H |  | one way anova | p<0.0001 | 1 |  |
| WT vs NALCN KO | 2H |  | Tukey post hoc | p<0.0001 |  |  |
| WT vs B-Arrestin KO | 2H |  | Tukey post hoc | p=0.9121 |  |  |
| WT vs GDP-BS internal | 2H |  | Tukey post hoc | p=0.0008 |  |  |
| Sodium leak current WTvsKO | 3A | WT(39);KO(20) | Mann-Whitney U-test | p<0.0001 | 0.99 |  |
| WT cell-att. Decrease in firing rate by dopamine puff in control conditions | 3E | 8 | Wilcoxon test | p=0.0078 | 0.99 |  |
| WT cell-att. Decrease in firing rate by dopamine puff in ba/TerQ | 3E | 8 | Wilcoxon test | p=0.0078 | 0.99 |  |
| WT W-C Decrease in firing rate by dopamine puff in control conditions | 3G | 9 | Wilcoxon test | p=0.0039 | 1 |  |
| WT W-C Decrease in firing rate by dopamine puff in ba/TerQ | 3G | 9 | Wilcoxon test | p=0.0039 | 0.99 |  |
| KO W-C Decrease in firing rate by dopamine puff in control conditions | 3I | 8 | Wilcoxon test | p=0.0039 | 1 |  |
| KO W-C Decrease in firing rate by dopamine puff in ba/TerQ | 3I | 8 | Wilcoxon test | p=0.078 | 0.17 |  |
| WT V-clamp baclofen 0.1 mM Calcium | 4A | 8 | RM-ANOVA | p<0.0001 | 1 |  |
| WT V-Clamp 2 vs 0.1 mM calcium | 4A | 8 | Tukey post hoc | p<0.0001 |  |  |
| WT V-Clamp 0.1 mM calcium vs baclofen | 4A | 8 | Tukey post hoc | p=0.0041 |  |  |
| WT V-Clamp 0.1 mM baclofen vs NMDG | 4A | 8 | Tukey post hoc | p=0.2102 |  |  |
| KO V-clamp baclofen 0.1 mM Calcium | 4B | 7 | RM-ANOVA |  |  |  |
| KO V-Clamp 2 vs 0.1 mM calcium | 4B | 7 | Tukey post hoc | p=0.3746 |  |  |
| KO V-Clamp 0.1 mM calcium vs baclofen | 4B | 7 | Tukey post hoc | p=0.8838 |  |  |
| KO V-Clamp 0.1 mM baclofen vs NMDG | 4B | 7 | Tukey post hoc | p>0.99 |  |  |
| Dopamine vs baclofen sensitive current | DA(14);Bclfn(8) | Mann-Whitney U-test | p=0.6642 | 0.1 |  |  |
| WT W-C Decrease in firing rate by dopamine puff in control conditions | | 5B | 8 | Wilcoxon test | p=0.0078 | 0.99 |
| WT W-C Decrease in firing rate by dopamine puff in ba/TerQ | 5B | 8 | Wilcoxon test | p=0.0078 | 0.99 |  |
| KO W-C Decrease in firing rate by dopamine puff in control conditions | 5D | 8 | Wilcoxon test | p=0.0078 | 0.99 |  |
| KO W-C Decrease in firing rate by dopamine puff in ba/TerQ | 5D | 8 | Wilcoxon test | p>0.99 | 0.05 |  |
|  |  |  |  |  |  |  |
|  |  |  |  |  |  |  |
| WT cell attached 300 nM baclofen wash | 6E | 7 | RM-ANOVA | p<0.0001 | 1 |  |
| WT cell attached control VS 300 nM baclofen wash | 6E | 7 | Tukey post hoc | p=0,0039 |  |  |
| WT cell attached baclofen VS CGP | 6E | 7 | Tukey post hoc | p=0,0032 |  |  |
|  |  |  |  |  |  |  |
